# Magnitude and pattern of improvement in processes of care for hospitalised children with diarrhoea and dehydration in Kenyan hospitals participating in a clinical network

**DOI:** 10.1111/tmi.13176

**Published:** 2018-11-11

**Authors:** Samuel Akech, Phillip Ayieko, Grace Irimu, Kasia Stepniewska, Mike English, Clinical Information Network authors

**Affiliations:** ^1^ Kenya Medical Research Institute/Wellcome Trust Research Programme Nairobi Kenya; ^2^ Department of Paediatrics and Child Health University of Nairobi Nairobi Kenya; ^3^ Centre for Tropical Medicine Nuffield Department of Clinical Medicine University of Oxford Oxford UK; ^4^ Worldwide Antimalarial Resistance Network Oxford UK

**Keywords:** children, dehydration, diarrhoea, hospitals, quality, diarrhée, déshydratation, qualité, enfants, hôpitaux

## Abstract

**Objective:**

WHO recommends optimisation of available interventions to reduce deaths of under‐five children with diarrhoea and dehydration (DD). Clinical networks may help improve practice across many hospitals but experience with such networks is scarce. We describe magnitude and patterns of changes in processes of care for children with DD over the first 3 years of a clinical network.

**Methods:**

Observational study involving children aged 2–59 months with DD admitted to 13 hospitals participating in the clinical network. Processes of individual patient care including agreement of assessment, diagnosis and treatment according to WHO guidelines were combined using the composite Paediatric Admission Quality of Care (PAQC) score (range 0–6).

**Results:**

Data from 7657 children were analysed and improvements in PAQC scores were observed. Predicted mean PAQC score for all the hospitals at enrolment was 59.8% (95% CI: 54.7, 64.9) but showed a wide variation (variance 10.7%, 95% CI: 5.8, 19.6). Overall mean PAQC score increased by 13.8% (95% CI: 8.7–18.9, SD between hospitals: ±8.2) in the first 12 months, with an average 0.9% (95% CI: 0.3–1.5, SD ± 1.0) increase per month and plateaued thereafter, and changes were similar in two groups of hospitals joining the network at different times.

**Conclusion:**

Adherence to guidelines for children admitted with DD can be improved through participation in a clinical network but improvement is limited, not uniform for all aspects of care and contexts and occurs early. Future research should address these issues.

## Abstract (fr)

**Objectif:**

L'OMS recommande d'optimiser les interventions disponibles pour réduire le nombre de décès d'enfants de moins de cinq ans souffrant de diarrhée et de déshydratation (DD). Les réseaux cliniques peuvent aider à améliorer les pratiques dans de nombreux hôpitaux, mais l'expérience de tels réseaux est rare. Nous décrivons l'ampleur et les profils des changements dans les processus de prise en charge des enfants souffrant de DD au cours des trois premières années d'un réseau clinique.

**Méthodes:**

Etude observationnelle chez les enfants âgés de 2 à 59 mois souffrant de DD et admis dans 13 hôpitaux participant au réseau clinique. Les processus de prise en charge individuelle des patients, y compris l'accord pour l'examen, le diagnostic et le traitement selon les directives de l'OMS, ont été combinés à l'aide du score composite (de 0 à 6) de la qualité des soins pédiatriques à l'admission (PAQC).

**Résultats:**

Les données de 7.657 enfants ont été analysées et des améliorations des scores PAQC ont été observées. Le score PAQC moyen prédit pour tous les hôpitaux au moment de l'inscription était de 59,8% (IC95%: 54,7‐64,9), mais a montré une grande variation (variance 10,7%; IC95%: 5,8‐19,6). Le score PAQC moyen global a augmenté de 13,8% (IC95%: 8,7‐18,9; DS entre hôpitaux: ± 8,2) au cours des 12 premiers mois, avec une augmentation moyenne mensuelle de 0,9% (IC95%: 0,3‐1,5; SD ± 1,0) pour ensuite atteindre un plateau et les changements ont été similaires dans deux groupes d'hôpitaux rejoignant le réseau à des moments différents.

**Conclusion:**

La participation à un réseau clinique peut améliorer l'adhésion aux directives pour les enfants admis avec DD, mais l'amélioration est limitée et non uniforme pour tous les aspects des soins et contextes et se produit tôt. Les recherches futures devraient aborder ces questions.

## Introduction

Diarrhoea and dehydration (DD) is still a major cause of death in children under 5 years, causing approximately 0.5 million deaths annually, mostly in sub‐Saharan Africa and Asia, and optimisation of available interventions has been identified as key to reducing case fatality over the next decade 1, 2, 3. WHO guidelines incorporate the best available evidence and have been adopted by Kenya's Ministry of Health as protocols for in‐hospital care 4, 5. However, guideline adoption at the national level does not always result in local implementation 6 that together amount to scaling up interventions. Thus, widespread adoption requires dissemination to all hospitals linked to active local efforts to promote guideline use. Clinical Networks are one means to promote more standardised approaches to care with examples of their value in some high‐income settings 7, 8. Employing some of the principles of existing clinical networks, 13 first‐referral level (district) hospitals in Kenya have been involved in a collaborative effort referred to as the Clinical Information Network (CIN). The Network has been aiming to improve the uptake of standard guidance for admission care for common diseases since October 2013 9. Wider benefits include the ability to better characterise paediatric admissions at scale and the use of shared data to implement and evaluate strategies that promote guideline adoption.

Major activities within the CIN include promoting the use of standardised patient forms (admission, treatment, and discharge) 10, regular audit and feedback on performance for chosen processes of care for common conditions, and network meetings 9, 11. The set of network activities employed drew on prior studies in Kenya 12, 13, an understanding of context and relevant theory. Studies report inconsistent effects, and therefore uncertain benefits, of such efforts to promote guideline adherence; for example, reviews report only modest improvements when audit and feedback (A&F) are used with median increases of 4.3% and 1.3% for indicators measured as dichotomous or continuous variables respectively 14.

A challenge for studies of whole networks is that typically there is no counterfactual enabling comparison of activities within a network to those outside it. Acknowledging this limitation, we use data collected by the CIN to describe changes in care provided to children with DD over the period hospitals have been engaged in this network. We used a composite score, the Paediatric Admission Quality of Care (PAQC) score, to describe the changes. The PAQC score summaries three domains of process of admission care (assessment, diagnosis and treatment of illnesses) into a single quality score for DD, malaria or pneumonia as discrete illnesses as well as when children have two or more of these illnesses 15.

We took advantage of the fact that hospitals joined CIN at different times, eight in late 2013 and five in early 2014, to consider ‘early’ and ‘late’ groups of hospitals (respectively), to consider the data from different before/after studies allowing us to compare the patterns of change in the two groups. We hypothesized that observing similar patterns of change would suggest that the effects of network activities were replicated across the two studies.

## Methods

### Study setting

We used data collected between October 2013 and November 2016 from 13 hospitals currently involved in CIN. CIN is a collaboration between Kenya's Ministry of Health (MOH), the Kenya Medical Research Institute/Welcome Trust Programme (KWTRP) and the Kenya Paediatric Association (KPA). Initial hospitals joined the network in October 2013 (eight hospitals, referred to as ‘early’ hospitals) while a second cohort joined in March 2014 (five hospitals, referred to as ‘late’ hospitals). The different entry periods were the result of logistical (largely geographical) considerations and hospitals were not randomly allocated. Within CIN, hospitals themselves are responsible for adopting and supplying recommended standard medical forms and care is provided entirely by the hospital personnel. The hospitals typically have a paediatrician but in some sites a general medical officer (a degree trained physician) to supervise other junior clinicians including medical officer interns, clinical officers (non‐degree trained physicians) and clinical officer interns in the absence of a paediatrician. Admission assessments and initial treatments are prescribed mostly by these junior clinicians with the team leader reviewing the management often 24–48 h later. New groups of intern clinicians rotate onto the paediatric wards every 3 months in all the hospitals with medical officer and clinical officer interns rotating at different timepoints. Treatment and other nursing care is provided by nurses and the hospitals often have shortages of nursing staff as is common in many resource poor settings.

We have previously described CIN processes in detail 9, 11. Briefly, information on admission, clinical information, immediate treatment and discharge information is abstracted into an online database by a specially trained clerk as soon as a child is discharged from hospital. This clerk is the only person at the hospital engaged by the research team. Data are then synchronised into a central server managed by KWTRP 16, 17. Reports on various processes of care are produced every 2–3 months and sent to the hospitals together with a combined report that summarises performance of all hospitals and allows them to compare their performance. Reports are delivered to each hospital both in print and soft copies for the paediatrician to disseminate, discuss with their clinical teams, and jointly identify action plans. The paediatricians received mentorship on how to give effective local feedback and how to develop a joint action plan with their teams. A senior paediatrician, based at KWTRP and coordinates CIN, calls the hospital paediatrician after each report and highlights key areas of improvement or deterioration. CIN meetings to share experience are held twice yearly and attended by the hospitals key personnel (paediatrician or designated clinician, nurse in charge of paediatric areas, and health records officer in charge). Studies using anonymised routine data from the CIN have been approved by the KEMRI scientific and ethics review unit.

### Defining diarrhoea with dehydration

We identified children in the database who: were admitted with a diagnosis of both diarrhoea and dehydration (DD); who were aged 2–59 months; had comprehensive information on assessment, diagnosis and inpatient management. We excluded children with bloody diarrhoea or with severe acute malnutrition (SAM), for whom there are different guidelines on managing dehydration, and children with a minimal dataset. The minimal dataset 9 consists of age, sex, diagnoses and outcome, which are required for routine health information system reporting. Such data are also collected for randomly selected records in two hospitals with high patient volumes, in all 13 hospitals for those admitted for surgery or burns and for all records when the clerk is on leave (total 4 weeks in a year).

### Definition of quality of care

The recently developed composite PAQC score measures multiple processes of care in children admitted with DD, malaria, or pneumonia separately or when these conditions co‐exist in the same individuals 15. The PAQC score measures three domains of the admission process of care: assessment, diagnosis and treatment of illnesses.

The assessment domain is made up of three items: recording of primary assessment signs required to diagnose the disease of interest, recording of any secondary signs necessary for disease severity classifications, and a third item representing complete documentation of all primary and secondary signs. The diagnosis domain is a single item representing whether a guideline recommended severity classification is indicated in the medical record; while the treatment domain is made up of two items, correct prescription and correct use of the treatment (dose, route, frequency and duration). The treatment domain for DD examines only correctness of fluid administration in accordance with WHO guidance for various severities of dehydration (shock, severe, some, none) and does not include use of zinc.

Each item has a binary score of 0/1 therefore the PAQC score ranges between 0 and 6. In children with DD plus pneumonia or malaria comorbidities, separate scores are estimated for each disease and then combined while preserving an overall patient level point score of 0–6 (this method is described in full elsewhere 15), facilitating reporting of quality in the presence of these common comorbidities. For these analyses individual PAQC scores were divided by 6 and multiplied by 100 to obtain a percentage score for each child and mean percentage PAQC scores were then calculated for all DD admissions to each hospital for each month.

### Analysis

We calculated the PAQC score for each patient and then created a binary measure to represent ‘good care’, defined as a score of five or six out of six. Using this categorical metric, we then calculated the proportion of patients with ‘good care’ for each month since joining the network. Month 1 is the month when a hospital joined, October 2013 or March 2014 for ‘early’ and ‘late’ groups respectively. We examined the changes in proportion with ‘good care’ from the baseline (month of joining the network). We also compared proportions with score of 3 or less, ‘good care’, or maximum score of six between the first and last 3 months in the network for each hospital.

Although CIN data are observational, we used the two phases of joining the network (‘early’ and ‘late’) to explore whether the pattern of response from time of joining the network (measured in months) was replicated across the two groups of hospitals. We hypothesised that observing such replication would provide some support for a generalisable effect of network participation on clinical practice.

Separate plots for the two groups of hospitals, drawn using mixed effects fractional polynomial regression, which allows for different baseline PAQC scores and changes over time across hospitals, showed that changes in PAQC score are bi‐phasic with a cut‐off at 12 months between the phases. Therefore, we fitted split‐line regression models to enable estimation of slopes (change in PAQC score over time) and intercepts (baseline PAQC scores) in the two phases. This was achieved by including a binary variable, coded as 0 for the initial phase and 1 for the second phase, in the model and included an interaction term for time in the network. PAQC scores for the two hospital groups were compared in this model by inclusion of a binary variable coding hospital type (early/late) and interaction term for time.

Similar analyses were undertaken to examine patterns of changes in specific process of care domains (assessment, diagnosis and treatment) to establish if different domains followed distinct trajectories with time. This latter analysis used combined data without categorisation into ‘early’ and ‘late’ hospitals. Analyses were done using Stata 13.1 18. A description of theory of change informing CIN 13, implementation strategies used 11 and how these might have resulted in changes seen have been published and were not the focus of this analysis.

## Results

We analysed data from 7 657 children admitted with DD including those with malaria and/or pneumonia comorbidity between October 2013 and November 2016, which comprised 16.0% (7 675/51 344) of the full dataset. Twelve and 11 feedback reports were provided for ‘early’ and ‘late’ hospitals, respectively, and six network meetings were held during this period. 44% (3 354/7 596) of participants were females, 57.5% (4 408/7 657) were infants (aged <1 year) and 3.3% (223/6 836) had diarrhoea duration longer than 14 days. Main secondary diagnoses (also referred to as comorbidities) were malaria 34.6% (2 646/7 657), pneumonia 37.5% (2 871/7 657), anaemia 4.2% (323/7 657) and clinically diagnosed meningitis in 4.4% (335/7 657).

**Figure 1:**
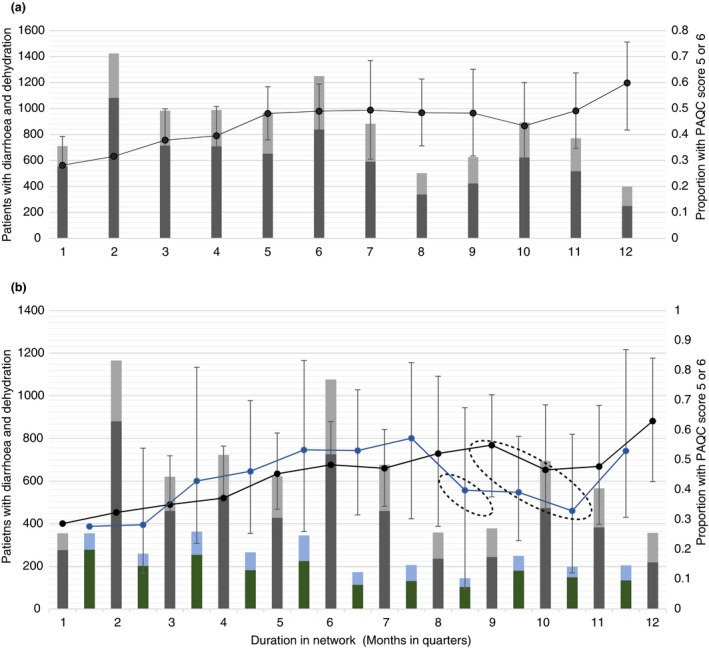
Patients with diarrhoea and dehydration receiving ‘good care’ over time in the network. Footnote: (a) Grey bars are the number of patients with Paediatric Admission Quality of Care (PAQC) scores = 5 or 6 (‘good care); black bars are the number of patients with PAQC score less than 5; maximum PAQC score = 6. (b) shows graphs for ‘early’ (first staked bar) and ‘late’ hospitals (second stacked bar); black and blue lines represent mean proportions and 95% confidence intervals for patients with ‘good care’ for ‘early’ and ‘late’ hospitals respectively; 1‐new intern in hospitals and all health worker strike in one hospital; 2‐new intern rotation, change in lead clinicians in two hospitals, and strikes in four hospitals. [Colour figure can be viewed at wileyonlinelibrary.com]

Figure 1 shows changes in proportions with ‘good care’ over the duration of participation in the network. Overall there was an improvement in the proportion of patients classified as receiving ‘good care’ but there were periods of fluctuations often coinciding with changes in clinical teams or health worker strikes.

Improvement in quality of care was observed on a number of indicators, when comparing first and last 3 months of the network for each hospital: the proportion of patients with perfect care (6 out of 6) increased from 17% (95% CI 11–25) to 46% (95% CI 29–64); the proportion of patients with ‘good care’ (5–6 out of 6) increased from 28% (95% CI 19–39) to 60% (95% CI 42–75); proportion of patients with poor care (≤3 out of 6) decreased from 37% (95% CI 27–47) to 16% (95% CI 8–29); overall the patient mean score increased from 63% (95% CI 58–69) to 75% (95% CI 67–83). Compared to the first 3 months, children admitted across network hospitals were more likely to get good care; the adjusted OR for hospitals was 4.5 (95% CI 2.9–6.9).

### Effect of network participation on processes of care for diarrhoea and dehydration

**Figure 2:**
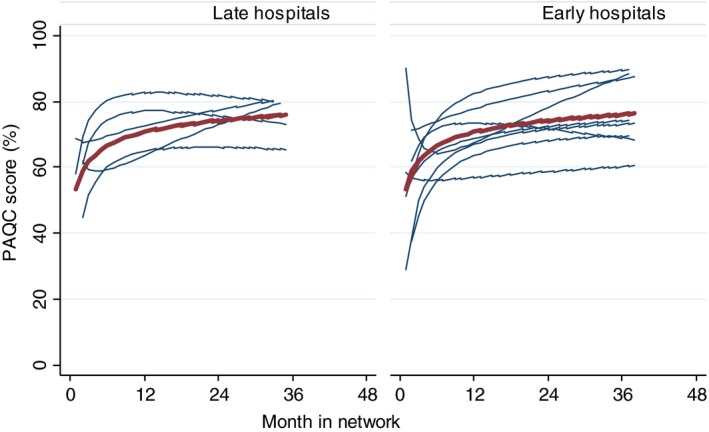
Predicted Paediatric Admission Quality of Care (PAQC) scores over time. Footnote: Left panel shows results for early entry hospitals (‘early hospitals’) and right panel shows results for late entry hospital (‘late hospitals’) Red line shows mean prediction assuming random effects equal to zero. The following mixed effects fractional polynomial model was fitted; paqc_score = 69.6 + 5.8 ln(duration_in_study/10)–0.6 ln^2^(duration_in_study/10) using this Stata command *‘*fp <duration_in_study>, scale: xtmixed paqc_score <duration_in_study> || hospitalID: <duration_in_study>‘. [Colour figure can be viewed at wileyonlinelibrary.com]

Lowess plots (not shown) consisting of individual PAQC scores created for each hospital showed a variety of patterns: (i) non‐linear change with a rapid initial increase and later plateau phase (7/13), (ii) sustained improvement throughout the duration of network participation (3/13), (iii) decline and improvement (2/13) and (iv) minimal change in performance over the entire period (1/13). The predicted trajectory showed biphasic improvement in PAQC scores with a rapid initial increase (first 12 months, Phase 1) and a plateau (Phase 2) afterwards in the network (Figure 2). The predicted mean percentage PAQC score for all hospitals at enrolment (first month) was 59.8% (95% CI: 54.7, 64.9), and was similar between early and late hospital groups (Table 1) but with wide variation across hospitals (variance 10.7% [95% CI: 5.8, 19.6]). The overall mean PAQC score increased by 13.8% (95% CI: 8.7–18.9, standard deviation between hospitals: ±8.2) in the first 12 months with an average 0.9% (95% CI: 0.3–1.5, SD ± 1.0) increase per month. Afterwards the change in score was not significant with a 0.1% (95% CI −0.2 to 0.3, SD ± 0.37) increase per month. There was no significant difference between early and late hospitals with respect to baseline PAQC scores and change in scores in the first or second phases (Table 1).

**Table 1:** Changes in Paediatric Admission Quality of Care (PAQC) scores and comparison between ‘early’ and ‘late’ hospitals

Parameter	PAQC score (%)	*P*‐value	95% CI
Baseline PAQC score at to network	59.8		54.7 to 64.8
Overall PAQC score at 12 months	73.6		67.0 to 80.2
Monthly increase in the first 12 months	0.9		0.3 to 1.5
Overall percentage increase in first 12 months	13.8		8.7 to 18.9
Monthly increase after 12 months	0.05		−0.2 to 0.3
Difference in monthly increase before and after 12 months	0.9		0.2 to 1.5
Difference between ‘early’ and ‘late’ hospitals			
Differences in baseline PAQC score at enrolment	−0.7	0.89	−10.9 to 9.5
Differences in PAQC score at 12 months	−4.0	0.43	−14.0 to 6.1
Differences in PAQC score increases in the first 12 months	−0.5	0.42	−1.7 to 0.7
Differences in PAQC score increases after 12 months	0.3	0.13	−0.1 to 0.8

### Patterns of change in specific processes of care

The mean monthly domain specific PAQC scores for all hospitals at baseline for assessment, treatment and diagnosis were 60.8% (95% CI: 34.5–87.0), 48.6% (95% CI: 33.8, 63.5) and 72.3% (95% CI: 50.0–94.6) respectively. Although mean scores improved in all domains, improvement varied widely between hospitals in all three domains during Phase 1 for assessment (overall mean increase = 8.4%, 95% CI: −4.6, 21.3), treatment (overall mean increase = 8.6%, 95% CI: 1.3, 15.9) and diagnosis (overall mean increase = 12.3%, 95% CI: −5.6, 30.1). Phase 2 showed slowed or stagnating changes in the assessment domain (overall mean increase = 0.8%, 95% CI: −2.4, 4.0) and a trend suggestive of more patients being identified as being incorrectly treated (overall mean increase = −1.3%, 95% CI: −3.5, 0.8), or that recommended classification was not used (overall mean increase = −6.2%, 95% CI: −11.9, −0.6).

## Discussion

Our analysis of data from 13 hospitals involved in collaborative efforts aimed at promoting guideline adherence across a range of common childhood illnesses in Kenyan hospitals shows that documentation for various processes of care improved 9, 11, 16, 17. Using a recently developed composite score for processes of admission care, the PAQC score 15, we show that the process of DD admission care as a whole improved over time. Most improvement was seen in the first year; subsequently changes in the PAQC score measured across hospitals slow down or plateau. Overall improvement occurs in the three domains of process of care (assessment, treatment, and diagnosis) but is greatest for the assessment domain. Hospitals have different performance at baseline and show different patterns of improvement in response to the network activities. This ‘hospital effect’ is perhaps not surprising given the complexity of hospitals as organisations and the reliance on local paediatric team leaders’ efforts to promote improved practice. It is also important to note that in each of these hospitals it is the junior clinicians who admit patients and whose practice is assessed with the data available in the clinical information network. These junior clinicians rotate regularly; hence in each hospital the junior staff changed on at least 8 occasions (3 monthly) during the study period. In several hospitals, the paediatric team leaders also changed but general improvements observed appear to be robust to such staff turnover.

Recent reviews demonstrate only modest improvement of compliance with practice recommendations when audit and feedback are used, namely a median increase of 4.3% for dichotomous and 1.3% for continuous outcomes 10. Our analysis suggests a mean improvement in PAQC score for children with DD of nearly 14% in the first 12 months and minimal change thereafter. We have previously shown some improvement in processes of in‐patient care through training of health workers, facilitation, evidence dissemination, supervision and face‐to‐face feedback derived from intermittent surveys in Kenya 6, 19, 20. In the current approach we sought to build on this platform and begin to explore the potential of clinical networks 21 linked to improved health information systems as a means to foster practice change. It has been suggested that networks may help overcome some of the ‘wicked problems’ inherent in achieving changes at scale, particularly if they can effectively engage sets of practice leaders who together embrace the change efforts 22, 23, 24.

This study raises questions on the nature of interventions that may be required as any change process evolves. Feedback in the network was given using methods associated with its effectiveness including provision by respected senior colleagues, frequent delivery, timeliness (2–3 monthly reports were based on data as recently as the previous week) and having both verbal and written components highlighting areas requiring improvement 14. After good improvements in the first year of activities, however, performance tended to stagnate. It is possible therefore that new strategies are required to motivate and facilitate continuous improvement.

Improvements in the diagnosis and treatment domains appeared harder to change, although for the diagnosis domain relatively high baseline performance may have led to a ceiling effect. It is possible therefore that feedback might be best suited for changing specific types of behaviour and that it may be less effective for more complex cognitive tasks that could also require elements of instruction or coaching at the individual level to foster improvement 14, 25, 26. In the current form of the network any coaching of individual junior clinicians is left to the paediatric team leader's discretion. Information systems that identify individual clinicians’ areas of weakness could offer a means to tailor the form of feedback to needs or help inform team leaders which aspects of the care process require attention.

Findings from the CIN network are limited as it only collects information on documentation of processes of care but we have shown that correct fluid prescription is associated with better survival in children with diarrhoea and dehydration 27. Our results therefore best represent clinicians’ intention to treat in a context where constraints in health personnel and supplies may exist. It is also not possible to disaggregate what activities of the network are effective limiting our ability to make recommendations for adoption of any single activity. Our study design does not allow us to make causal claims that CIN activities promote guideline adoption. The efforts of networks are hard to examine using experimental designs as identifying a counterfactual population is challenging and networks themselves are typically a *n* = 1 activity (while not necessarily being an entirely homogenous intervention within all participating sites). Time series evaluations (including statistical process control methods) might have provided more credible evidence of effects than the before/after analyses we conducted. However, there are difficulties conducting such studies where the information available prior to intervention may be poor (and indeed one aim of the network was to improve such information). Additional qualitative research does support the proposition, the CIN was valuable in promoting changes in practice and guideline adoption through pastoral practices 28; however questions remain over whether this strategy would work in all contexts.

In this analysis we only present quantitative outcome of processes of care for one condition but we recognise that CIN intervention was implemented in complex adaptive system and a realistic evaluation examining the context, mechanisms and outcomes would be most appropriate 29. However, we have previously described theory of change informing CIN intervention 13, implementation strategies adopted 11 using typology described by Powell *et al* [30.], including which components of implementation strategies fall in various domains of realistic evaluation 11.

## Conclusions

Care of children admitted with DD can be improved by a set of activities encompassed in a clinical network. Future research should explore how audit, feedback and linked interventions can best support changes for different contexts, for different tasks, at different stages of the performance cycle, and define appropriate implementation strategies for achieving these.

## Clinical Information Network authors

The Clinical Information Network (CIN) team who contributed to this work as part of the CIN author group includes: Samuel Ngarngar (Vihiga County Hospital), Nick Aduro & Ivan Injira (Kakamega County Hospital), Loice Mutai, Christine Manyasi & David Kimutai (Mbagathi County Hospital), Caren Emadau, Cecilia Mutiso & Celia Muturi (Mama Lucy Kibaki County Hospital), Charles Nzioki & SupaTunje (Machakos County Hospital), Francis Kanyingi & Agnes Mithamo (Nyeri County Hospital), Magdalene Kuria (Kisumu East County Hospital), Samuel Otido (Embu County Hospital), Alice Kariuki & Grace Wachira (Karatina County Hospital), Peris Njiiri (Kerugoya County Hospital), Rachel Inginia & Melab Musabi (Kitale County Hospital), Barnabas Kigen & Sande Charo (Busia County Hospital), Grace Akech Ochieng & Lydia Thuranira (Kiambu County Hospital), Morris Ogero; Thomas Julius; Mercy Chepkirui& James Wafula (KEMRI‐Wellcome Trust Research Programme).

## Funding

Funds from The Wellcome Trust (#097170) awarded to ME as a Senior Fellowship together with additional funds from a Wellcome Trust core grant awarded to the KEMRI‐Wellcome Trust Research Programme (#092654) supported this work. SA is supported by the Initiative to Develop African Research Leaders (IDeAL) Wellcome Trust award (#107769/Z/15/Z). The funders had no role in drafting or submitting this manuscript.

## Ethics approval

KEMRI Scientific and Ethical Review Committee approved the CIN study. The Kenya Ministry of Health gave permission for this work to be done but no individual patient consent was obtained.

## Data sharing statement

Data for this report are under the primary jurisdiction of the Ministry of Health in Kenya. Enquiries about using the data can be made to the KEMRI‐Wellcome Trust Research Programme Data Governance Committee. The authors had full access to all data in the study and take responsibility for the integrity of the data and the accuracy of data analysis.
